# Assessment of quantification accuracy and image quality of a full‐body dual‐layer spectral CT system

**DOI:** 10.1002/acm2.12243

**Published:** 2017-12-20

**Authors:** Sebastian Ehn, Thorsten Sellerer, Daniela Muenzel, Alexander A. Fingerle, Felix Kopp, Manuela Duda, Kai Mei, Bernhard Renger, Julia Herzen, Julia Dangelmaier, Benedikt J. Schwaiger, Andreas Sauter, Isabelle Riederer, Martin Renz, Rickmer Braren, Ernst J. Rummeny, Franz Pfeiffer, Peter B. Noël

**Affiliations:** ^1^ Chair of Biomedical Physics Department of Physics and Munich School of BioEngineering Technical University of Munich Garching Germany; ^2^ Department of diagnostic and interventional Radiology Technical University of Munich Munich Germany

**Keywords:** dual‐energy CT, effective atomic number, iodine quantification, spectral CT, virtual mono‐energetic imaging

## Abstract

The performance of a recently introduced spectral computed tomography system based on a dual‐layer detector has been investigated. A semi‐anthropomorphic abdomen phantom for CT performance evaluation was imaged on the dual‐layer spectral CT at different radiation exposure levels (CTDI_vol_ of 10 mGy, 20 mGy and 30 mGy). The phantom was equipped with specific low‐contrast and tissue‐equivalent inserts including water‐, adipose‐, muscle‐, liver‐, bone‐like materials and a variation in iodine concentrations. Additionally, the phantom size was varied using different extension rings to simulate different patient sizes. Contrast‐to‐noise (CNR) ratio over the range of available virtual mono‐energetic images (VMI) and the quantitative accuracy of VMI Hounsfield Units (HU), effective‐Z maps and iodine concentrations have been evaluated. Central and peripheral locations in the field‐of‐view have been examined. For all evaluated imaging tasks the results are within the calculated theoretical range of the tissue‐equivalent inserts. Especially at low energies, the CNR in VMIs could be boosted by up to 330% with respect to conventional images using iDose/spectral reconstructions at level 0. The mean bias found in effective‐Z maps and iodine concentrations averaged over all exposure levels and phantom sizes was 1.9% (eff. Z) and 3.4% (iodine). Only small variations were observed with increasing phantom size (+3%) while the bias was nearly independent of the exposure level (±0.2%). Therefore, dual‐layer detector based CT offers high quantitative accuracy of spectral images over the complete field‐of‐view without any compromise in radiation dose or diagnostic image quality.

## 1 INTRODUCTION

Computed tomography (CT) is widely used in diagnostic imaging. Many of today's design considerations in state‐of‐the‐art CT systems provide a reduction in radiation exposure and enhance contrast‐to‐dose efficiency while increasing the perceived contrast‐to‐noise‐ratio. Among these developments, advanced iterative reconstruction techniques are a very promising tool.^1^, ^2^, ^3^, ^4^ However, despite these advances, extracting accurate information from CT images like object size and composition is still a challenge. Over the last years, spectral imaging methods using different dual‐energy approaches (kVp switching,^5^ dual x‐ray sources^6^) have attracted increased attention in research and clinical practice. Besides CT, also interventional 2D imaging techniques are foreseen to benefit from spectral imaging thechniques.^7^, ^8^ Those methods enable the quantification of object composition by exploiting measurements of the material‐ and energy‐dependent x‐ray attenuation of various materials using a low and high energy spectrum. Despite dual‐energy CT having been proposed shortly after the invention of CT itself,^9^, ^10^ clinically relevant systems have only been available for the recent years. Physically, the linear x‐ray attenuation coefficient μ that is reconstructed in each voxel of a conventional CT is a function of the x‐ray energy E, the material composition in the voxel represented by the involved atomic numbers Z and the mass density ρ in the voxel. A parametrization that is commonly used in diagnostic imaging expresses the total linear attenuation as the sum of photoelectric absorption and Compton effect^9^

(1) $$\mu \left(E,Z,\rho \right)=\left(a_{\mathrm{ph}}\left(Z\right)f_{\mathrm{ph}}\left(E\right)+a_{C}\left(Z\right)f_{C}\left(E\right)\right)\cdot \rho$$

In the above equation, $f_{\mathrm{ph}}\left(E\right)$ and $f_{C}\left(E\right)$ are the energy‐dependent spectral basis functions for the photoelectric absorption and Compton effect while $a_{\mathrm{ph}}\left(Z\right)$ and $a_{C}\left(Z\right)$ provide material‐specific weighting factors for both contributions. Therefore, the reconstructed HU‐values in conventional CT can be the same for two different types of materials. A very common and descriptive example for this is the discrimination of iodinated blood and calcified plaques in contrast‐enhanced CT angiography. Despite a large difference in the atomic number of these two materials, the reconstructed HU values may be identical depending on the local mass density or concentration of dissolved material. Using dual‐energy spectral CT, this limitation can be overcome by measuring the attenuation at two distinct energy levels $E_{L}$ and $E_{H}$. Substituting these energies into (eq. (1)) forms a set of two equations in two unknown variables and can be numerically solved for the underlying material composition prior to or following the CT reconstruction. Such effective energy levels can in principle be obtained by measuring the quality of the x‐ray beam using known calibration objects. Although this approach is only exactly valid in case of monochromatic energies $E_{L}$ and $\;E_{H}$, it can be extended to the case of polychromatic spectra such as those used in CT scanners.

With previously existing dual‐energy CT, a range of applications have been developed.^11^, ^12^ Virtual mono‐energetic imaging^13^ offers images with an appearance as if they were acquired with a mono‐energetic x‐ray beam, thereby reducing beam hardening artifacts,^14^ increasing image contrast between lesions and healthy parenchyma^15^ and potentially reducing radiation exposure.^16^ Furthermore, dual‐energy based material separation can be utilized to quantify contrast medium uptake and to obtain virtual noncontrast material–enhanced images.^17^ E.g., iodine density maps^18^ are applied in CTA, lesion characterization,^19^ and lung perfusion assessment.^20^ Besides these applications, many other clinical applications of material‐specific imaging have been identified in recent years.^21^, ^22^, ^23^, ^24^ In future and with the introduction of fully spectral CT systems it can be expected to define additional clinical applications, for example in the area of oncological imaging of therapy response.

The scope of this study is to characterize a novel type spectral CT system which is based on a dual‐layer detector. Potential advantages of dual‐layer CT include complete spatial and temporal registration of the acquired datasets combined with high temporal resolution and permanent recording of dual‐energy data in every scan facilitated by the detector design. Using detailed studies of the quantitative imaging performance, we are aiming to assess the usability of clinical dual‐layer CT in diagnosis of the various diseases mentioned above. Following an overview over the used CT system, we present measurements performed with an anthropomorphic phantom mimicking various types of body tissues and iodine concentrations that are typically encountered in clinical radiology. In detail, evaluation of CNR enhancement in a low contrast phantom using virtual mono‐energetic images (VMIs) at multiple energy levels, the quantitative accuracy of Hounsfield Units (HU) values for different tissue equivalent materials in VMIs and the quantitative accuracy of iodine‐concentration and effective‐Z measurements have been studied.

## 2 MATERIALS AND METHODS

### 2.A Dual‐layer spectral CT

**Figure 1:**
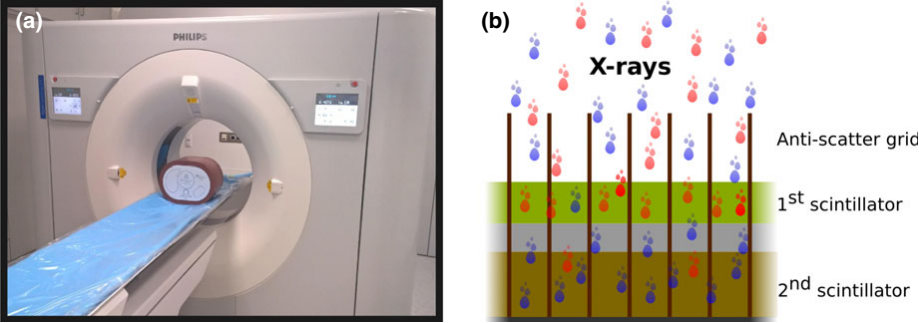
The spectral CT scanner used for this study. (a) shows a photograph of the dual‐layer spectral CT system installed at the Klinikum Rechts der Isar of the Technical University of Munich, Germany. (b) outlines the principle of the dual‐layer detector system. A polychromatic beam of x‐rays impinges on the detector. The low energy photons (represented by red dots) are mainly absorbed by the top scintillator which is closer to the x‐ray source. This first layer is mostly transparent to higher energy photons (represented by blue dots), which are then registered by a strongly absorbing second scintillator.

The experiments were carried out on a commercially available dual‐layer spectral CT (IQon spectral CT, Philips Healthcare, Cleveland, OH, USA). This novel scanner acquires spectral data per default at each CT scan, exploiting a dedicated dual‐layer detector concept.^25^, ^26^, ^27^ In contrast, other current dual‐energy CT systems can only acquire spectral information with preselected protocols. Figure 1(a) shows a picture of the dual‐layer spectral CT system along with a schematic drawing of the detector principle in Fig. 1(b).

The used detector concept employs two separate scintillator layers and readout electronics stacked on top of each other. Material and thickness of each layer are optimized such that the top layer mainly absorbs low‐energy photons while the high‐energy part is registered by the bottom layer. A crucial challenge for current dual‐layer CT is the relatively large overlap between low‐ and high‐energy spectra.^28^, ^29^ This limitation, however, can be overcome by carefully modeling the imaging process based on the physical properties of the complete CT system, including the x‐ray source, the interaction of the x‐ray beam with the patient and the detector properties to facilitate material decomposition.^30^ By design of the system, the spectral data are acquired simultaneously and perfectly registered in the projection domain. This enables the use of projection‐based material decomposition which allows for efficient correction of beam‐hardening artifacts and highly efficient noise reduction in the reconstructed images.^11^, ^25^, ^26^, ^31^ Along with the spectral image data, conventional polychromatic images are always obtained by summing up the low and high energy channels in the projection space. Exploiting the dual‐layer concept, the scanner offers dual‐energy data acquisitions per default in every CT scan. Therefore, there is no need for selecting a specific dual‐energy protocol prior to an examination and all data can be processed with any kind of spectral analysis offered by the system in retrospective.

### 2.B Description of phantoms

**Figure 2:**
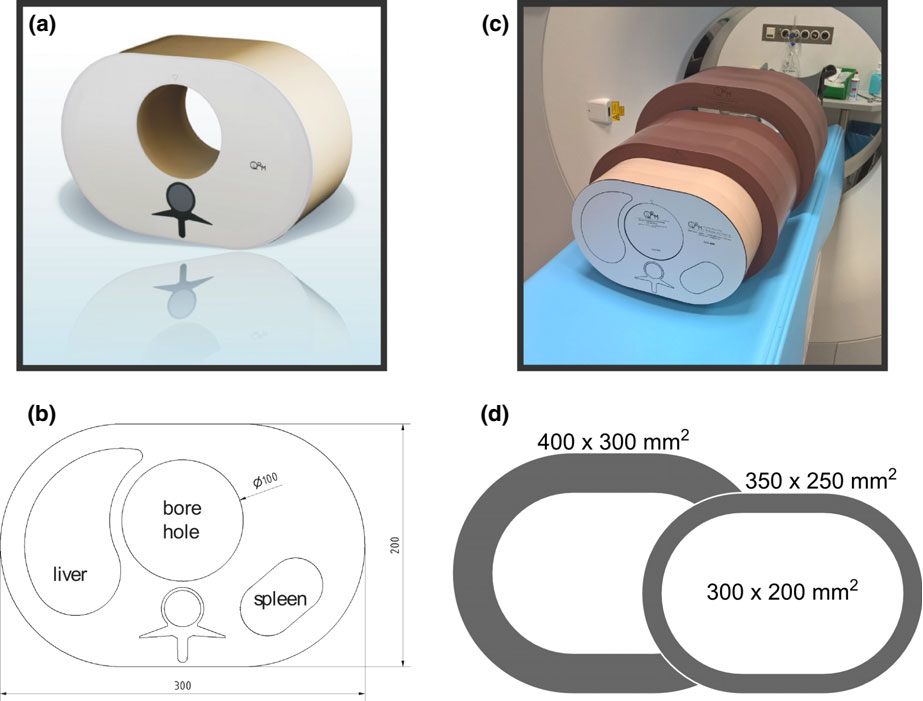
Images of the semi‐anthropomorphic abdominal phantom used to assess the image quality of the dual‐layer spectral CT scanner. The phantom features soft‐tissue (35 HU), liver, spleen (both 55 HU), and spinal contrast features and can be extended with task‐specific inserts into the central borehole (a). The CT numbers mentioned for the phantom features are reference values valid for a tube voltage of 120 kVp. The phantom can be extended to mimic obese patients using additional extension rings outside the basic phantom (b). The dimensions of the phantom in (c) are all given in mm.

To assess the image quality, a semi‐anthropomorphic abdomen phantom was used (QRM‐Abdomen, QRM GmbH, Moehrendorf, Germany). For an overview of the used phantom see Fig. 2. The phantom features objects representing tissues from liver, spleen, and the vertebral column, Figs. 2(a) and 2(b). The original dimensions of the phantom are 300 × 200 mm^2^ while larger and obese patients can be simulated using extension rings of tissue‐equivalent material. For the presented studies, a medium extension ring (350 × 250 mm^2^) and a large ring (400 × 300 mm^2^) consisting of fat‐like material was used in addition to the basic phantom as indicated in Figs. 2(c) and 2(d).

**Figure 3:**
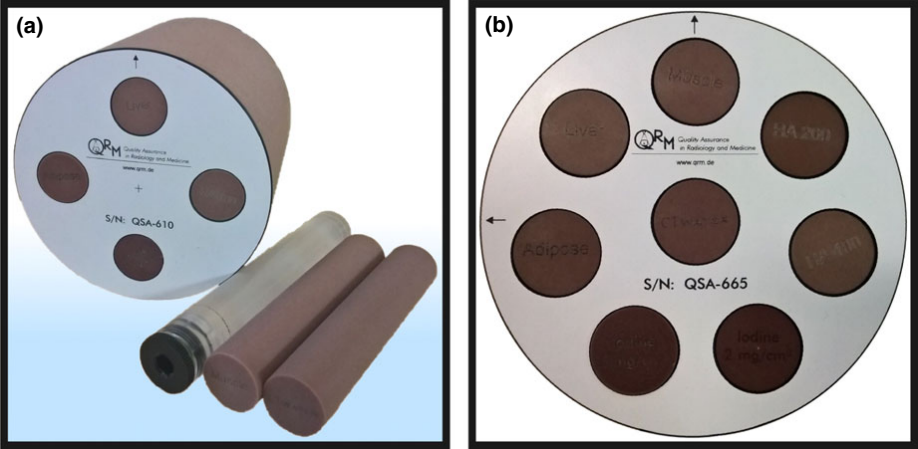
Images of the custom dual‐energy phantom insert with 100 mm diameter (a) and the specific configuration used to assess the CNR in VMIs (b). The background of the insert consists of water‐equivalent material (app. 0 HU at 120 kVp). The dual‐energy phantom features different rods made from tissue surrogate material as well as varying concentrations of iodine and Ca‐Hydroxyapatite. Fillable inserts are additionally provided for customized applications.

A central 100 mm borehole in the abdomen phantom allows the installation of task‐specific inserts used to assess the performance of a CT system with respect to special questions (see Fig. 3).

To study the quantitative accuracy of HU values and CNR in VMIs, the iodine‐water decomposition as well as effective‐*Z* values, a custom‐made dual‐energy phantom from the same manufacturer was used which provides four additional slots for selectable inserts. Available materials are listed in Table 1.

**Table 1:** Insert rods for the customized dual‐energy phantom. Along with a description of the tissue equivalents, the table lists the approximate CT numbers for each material at 120 kVp tube voltage. The iodine concentrations are embedded in solid water‐equivalent plastic rods

Material equivalent insert	Nominal elemental composition (%)								Density (g/cm^3^)	App. HU
	H	C	N	O	Cl	Ca	P	I		
Water	9.03	45.17	30.63	13.35	–	1.82	–	–	1.015	0
Muscle	8.93	45.11	30.82	13.08	–	2.05	–	–	1.057	44
Liver	8.92	45.12	30.89	13.00	–	2.07	–	–	1.065	54
Adipose	9.49	46.67	31.88	11.96	–	–	–	–	0.970	–80
Bone, BMD 200 mg/ml	7.60	34.23	34.17	12.54	–	8.31	3.15	–	1.158	255
Bone, BMD 400 mg/ml	6.39	28.62	28.57	17.27	–	13.49	5.66	–	1.288	502
Iodine 0.5 mg/ml	8.51	62.67	11.44	15.20	0.14	1.99	–	0.05	1.035	18
Iodine 0.75 mg/ml	8.50	62.66	11.44	15.20	0.14	1.99	–	0.07	1.036	28
Iodine 1.0 mg/ml	8.50	62.64	11.43	15.19	0.14	1.99	–	0.10	1.037	34
Iodine 2.0 mg/ml	8.48	62.63	11.41	15.16	0.14	1.99	–	0.19	1.033	60
Iodine 5.0 mg/ml	8.44	62.52	11.35	15.08	0.14	1.98	–	0.48	1.037	128
Iodine 10.0 mg/ml	8.38	62.32	11.27	14.95	0.14	1.95	–	1.00	1.039	266
Iodine 15.0 mg/ml	8.31	62.15	11.17	14.85	0.14	1.95	–	1.44	1.040	388

### 2.C Data acquisition and image reconstruction

The image acquisition and reconstruction was carried out using standard clinical protocols. An abdominal protocol was employed with the details given in Table 2. Images were obtained using varying dose levels, without using an exposure modulation to keep the dose constant between different phantom inserts. The conventional images were reconstructed from contributions of both detector layers using the proprietary *iDose4* reconstruction algorithm at level 0, corresponding closely to a FBP reconstruction with a minimum of additional iterative postprocessing. Additionally, spectral basis image (SBI) datasets were reconstructed for each scan. The spectral reconstruction algorithm offers the same levels for iterative postprocessing of reconstructed CT images as the iDose algorithm. Therefore, spectral level 0 was used to reconstruct data with minimum postprocessing (smoothing) of the reconstruction. As mentioned earlier, the spectral results generated by the dual‐layer CT exploit the anticorrelated behavior of noise to reduce the overall noise level in spectral basis images. However, it should be noted that this step mainly acts directly on acquired projection data^32^ and therefore does not involve a postprocessing of reconstructed CT images. The slice thickness was chosen to be 3 mm at a slice spacing of 3 mm.

**Table 2:** Acquisition parameters for the abdominal scan protocol used during the study

Protocol name	kV	Mean CTDI_vol_	Collimation (mm)	Slice width (mm)	Reconstruction
Abdomen spiral	120	10/20/30	64 × 0.625	3	iDose/spectral, level 0

All measurements have been taken three different dose levels with a CTDI_vol_ of 10 mGy, 20 mGy and 30 mGy per image. These exposure levels were chosen to reflect the diagnostic reference value of 25 mGy for adult abdominal CT set by the American College of Radiology. Additionally each single scan was repeated five times to reduce the influence of statistical effects on the reconstruction results.

**Figure 4:**
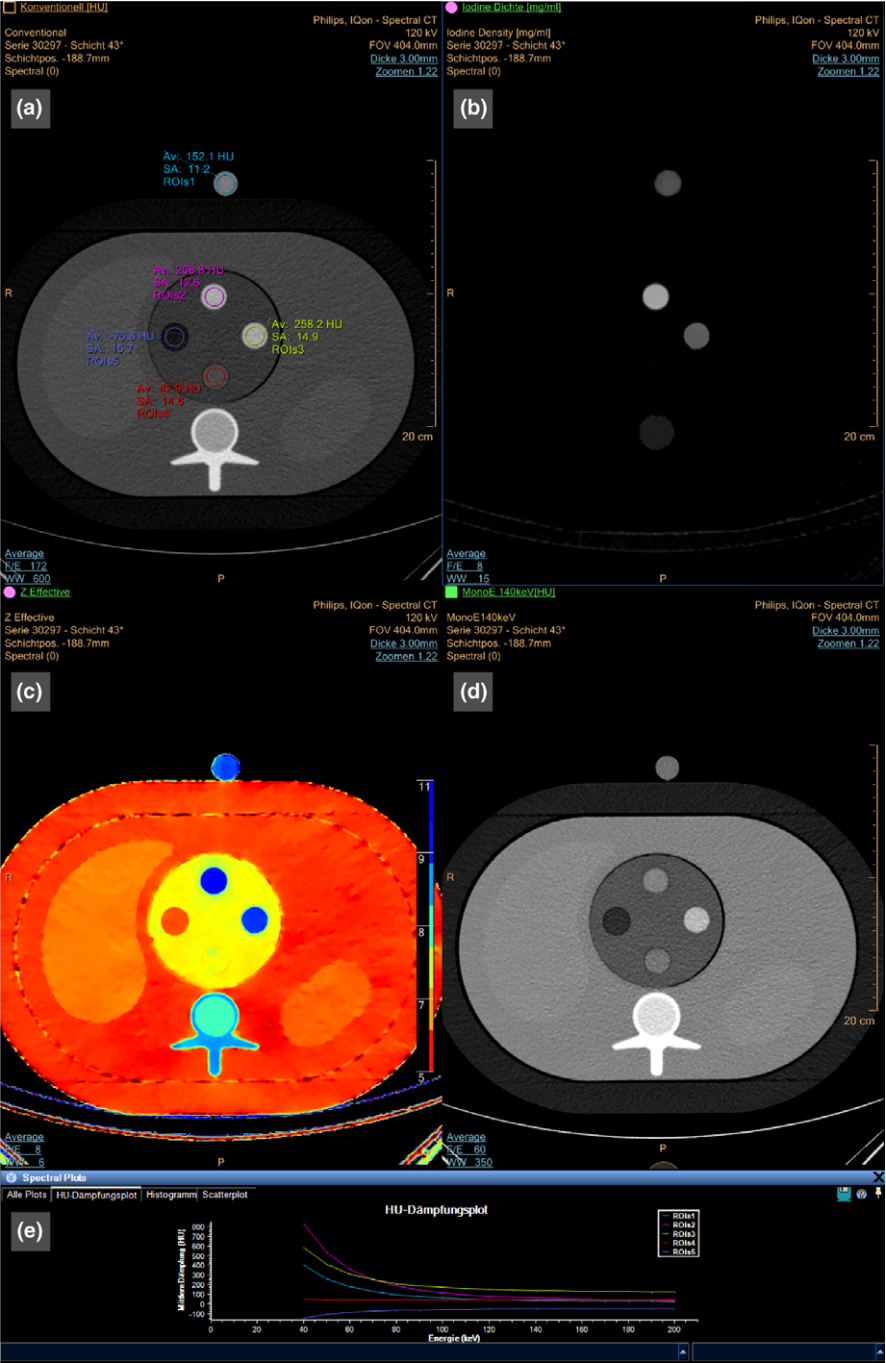
Illustration of the spectral CT evaluation workflow using a commercial spectral viewer. Different selectable spectral results are shown by the toolbox. Here, the conventional image (a), an iodine density‐map (b), an effective‐Z map (c), and a virtual mono‐energetic image (d) are shown. Additionally, energy‐dependent attenuation values in the HU scale can be determined for each selected region of interest (ROI) (e).

The exact type of the spectral images (virtual mono‐energetic, iodine maps, etc.) was chosen task‐specifically after the reconstruction itself. This was done using the spectral CT viewing tools integrated into a commercial software (IntelliSpace Portal V6.5.0.02901, Philips Healthcare, USA). An example of spectral results generated by this toolbox is provided in Fig. 4, illustrating the typical workflow of the spectral CT evaluation steps described in this paper.

### 2.D Measurements of CNR in virtual mono‐energetic images

**Figure 5:**
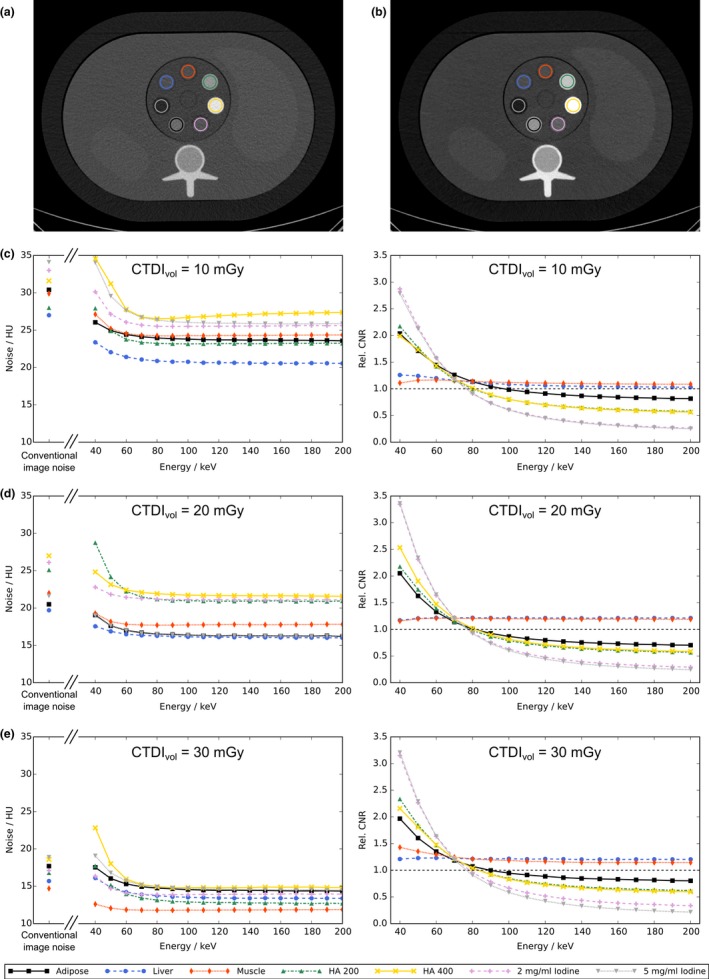
Measured contrast‐to‐noise (CNR) curves of the medium‐sized phantom (350 × 250 mm^2^) at various energy levels. (a) is the conventional CT image acquired using 120 kVp while (b) depicts the same slice at a 50 keV VMI derived from the spectral data. The curves in (c–e) show the energy‐dependent CNR and noise values in the virtual mono‐energetic images (VMI) for CTDI_vol_ ranging from 10 mGy to 30 mGy. The left column of plots in (c–e) gives the energy‐dependent behavior of image noise in terms of the HU standard deviation measured in each ROI. From these curves it can be seen that the noise is kept at a constant level which is lower than the conventional reference value for energy levels greater than 50 keV. Since the observed difference in HU values between two materials typically increases toward lower energies, increased CNR can be observed for all contrasts compared to the conventional image. The gain in CNR is most evident for materials with a larger difference in spectral behavior, yielding a more than threefold increase compared to the conventional reference image. Therefore, the increased CNR can mostly be attributed to the increased HU difference in the VMIs.

To study the behavior of contrast‐to‐noise ratio in spectral images, virtual mono‐energetic images at multiple energy levels exploiting the complete available energy range of 40 keV–200 keV were reconstructed in steps of 10 keV. The standard deviation of reconstructed voxel values was used as a measure of image noise. To determine the mean HU values and standard deviations, a circular region of interest (ROI) of 220 mm^2^ was selected in the tissue‐equivalent image features of the phantom and in the background as depicted in Figs. 5(a) and 5(b). For each energy level, the CNR relative to the background was calculated according to the following equation:

(2) $$\mathrm{CNR}\left(E\right)=\frac{\left|{\bar{\mathrm{HU}}}_{c}\left(E\right)-{\bar{\mathrm{HU}}}_{\mathrm{bg}}\left(E\right)\right|}{\sqrt{\sigma_{c}^{2}\left(E\right)+\sigma_{\mathrm{bg}}^{2}\left(E\right)}},$$

where E is the photon energy, ${\bar{\mathrm{HU}}}_{c}$ and ${\bar{\mathrm{HU}}}_{\mathrm{bg}}$ are the mean values of the ROIs in the contrasting features and the background and σ are the associated standard deviations.

The obtained CNR values were normalized to the respective values in the conventional full‐spectrum images to enable direct comparison between spectral and conventional reconstructions. The CNR was evaluated for the medium‐sized phantoms representing an average patient collective.

### 2.E Quantitative assessment of HU accuracy in virtual mono‐energetic images

HU are in general an energy‐dependent quantity due to the different behavior of the attenuation of body tissues compared to water. For higher x‐ray energies of 80 keV and more, this dependency, however, becomes very weak and can be neglected when working with polychromatic tube sources. In VMI, however, the change in HU values becomes evident when approaching lower energies and image readers might deal with considerably different HU values for certain tissue types.

The quantitative accuracy of the synthesized VMIs was investigated by comparing the values obtained in the above mentioned phantom measurement with theoretically calculated HU values for each energy. Using the chemical composition and mass densities of each feature, the theoretical HU value can be calculated from tabulated attenuation coefficients:

(3) $$\mathrm{HU}\left(E\right)=1000\cdot \frac{\rho_{\mathrm{mat}}\cdot \sum_{i}a_{i}{\left(\mu (E)/\rho \right)}_{i}-\mu {\left(E\right)}_{H_{2}O}}{\mu {\left(E\right)}_{H_{2}O}},$$

where $\rho_{\mathrm{mat}}$ is the mass density of the material of interest, the index i identifies each chemical element in the feature $a_{i}$ is the associated mass fraction and ${\left(\mu \left(E\right)/\rho \right)}_{i}$ the corresponding mass attenuation coefficient tabulated e.g., in the XCOM database.^33^ Finally, $\mu {\left(E\right)}_{H_{2}O}$ describes the attenuation of pure water at energy level *E*.

Using the spectral software, energy‐dependent attenuation plots were generated for each selected ROI in the energy range between 40 keV and 200 keV. These values were compared to the theoretical reference obtained using (eq. (4)) with compositions of each material reported by the phantom manufacturer. Since the mass density values and chemical compositions for each material have been measured at phantom production by the manufacturer these values are subject to fluctuations. Therefore, error intervals ΔHU(*E*) have been determined for each nominal value by applying error propagation to (eq. (4)) and inserting the fabrication tolerances reported by the manufacturer:

(4) $$\Delta \mathrm{HU}\left(E\right)=\sqrt{\sum_{i}{\left|\frac{\partial \mathrm{HU}(E)}{\partial x_{i}}\right|}^{2}\cdot \Delta x_{i}^{2}.}$$

Tolerances in the range of ± 20 HU are obtained for the calculated theoretical values.

HU values were measured within the medium‐sized phantom.

### 2.F Quantitative assessment of iodine concentrations in spectral images:

Currently, a majority of all CT studies involve contrast agents (nonionic iodine), with average volumes of 1 ml/kg body weight per study.^34^, ^35^ For the quantification of iodine concentrations, the dual‐layer spectral CT scanner offers the possibility to generate different iodine‐maps. In a first map, called the iodine‐no‐water image, the presumed concentration of iodine in mg/ml is calculated for each voxel. Voxels with a concentration well below 0.5 mg/ml iodine are assumed to contain only water‐like material and are suppressed in the image.^36^ However, the iodine‐no‐water image may also contain contributions arising from calcium, for example in bone or calcified plaques. Therefore, a second iodine‐map may be generated where the knowledge of the unique spectral dependence of the attenuation of each material is exploited to distinguish iodine from any other material. Such images are named iodine‐density maps since every material but iodine is suppressed in the images.

To investigate the accuracy of the iodine quantification in spectral reconstructions, the phantom was equipped with different rods containing various known concentrations of iodine in a water‐equivalent material (Table 1). For the quantification of voxels at the phantom periphery, iodine samples have been attached to the lateral outside surface of the phantom body to study eventual field‐of‐view (FOV)‐dependent effects in the decomposition accuracy.

Since the quantification of iodine content is considered to be of great clinical interest,^37^ the accuracy of this spectral results have also been determined in dependency on the patient size. Additional measurements have been performed with the basic phantom without additional fat‐like material and using the 400 × 300 mm^2^ extension ring to simulate large/obese patients.

### 2.G Quantitative assessment of the effective‐Z accuracy:

Effective atomic numbers obtained by the dual‐layer spectral CT are compared to theoretical reference values calculated from the nominal phantom composition. Using the tissue‐equivalent materials as well as iodine inserts for the customized dual‐energy phantom, the effective‐Z values were calculated using

(5) $$Z_{\mathrm{eff}}=\;\sqrt[{2.94}]{\sum_{i}f_{i}Z_{i}^{2.94}},$$

where i identifies every chemical element contained in the insert, $f_{i}$ is the associated fractions of electrons contributed by element i and $Z_{i}$ the corresponding atomic number.^38^ In the case of effective atomic numbers, the medium‐sized phantom configuration was used again.

## 3 RESULTS

### 3.A Measurements of CNR in virtual mono‐energetic images:

The results from the energy‐dependent CNR determination are summarized in Fig. 5. The panels A and B show a conventional CT image and a 50 keV VMI taken at the medium exposure level using the same widow and level settings. The increased image contrast and altered CT numbers are clearly to be seen in the low‐energy VMI. The ROIs for which the CNR was evaluated are marked in both panels. The left column of Figs. 5(c)– 5(e) shows the behavior of measured image noise in conventional reconstructions and VMIs. For energies greater than 50 keV, all investigated tissue substitutes showed smaller noise levels in VMIs compared to conventional reconstructions. Furthermore, the noise level becomes almost independent of energy above 70 keV. The right column of plots presents the obtained CNR values normalized to the values found in the conventional images. Toward lower energy levels, the CNR observed in VMIs significantly exceeds the values in conventional images for all materials. At 70 keV the mean CNR increase over all materials and dose levels was 20% while at 50 keV, a 74% improvement was obtained over all materials and dose levels. For materials with strongly different spectral behavior compared to the water‐equivalent background, a more than three‐fold CNR boost could be obtained in the case of iodine at 40 keV.

The results presented in Fig. 5 can be understood by comparing the normalized CNR curves to the curves of the image noise at the corresponding energy levels. Exploiting the characteristic anticorrelated noise properties of the spectral base images,^25^, ^39^ the resulting noise in the VMIs can be kept at nearly constant values which are below the noise levels in the conventional CT image. Only toward very low energies, a slight increase in the noise can be observed (left column of Figs. 5(c)–5(e). Thereby, the improved CNR is dominated by the boosted contrast between different tissue types when the energy is decreased. For tissue types with spectral characteristics similar to the water‐equivalent background in the phantom (e.g., liver and muscle) both the image contrast and the noise levels in VMIs become nearly independent of energy. Therefore, CNR curves for such materials become almost flat and are determined mainly by the lowered noise levels, leading to increased CNR over the complete energy range. At the chosen reconstruction settings, VMIs lead to an overall improved CNR especially at lower energies. This could be observed for a broad range of HU contrasts and exposure levels exploiting the decreased and flattened noise levels in VMIs.

### 3.B Quantitative assessment of HU accuracy in virtual mono‐energetic images:

**Figure 6:**
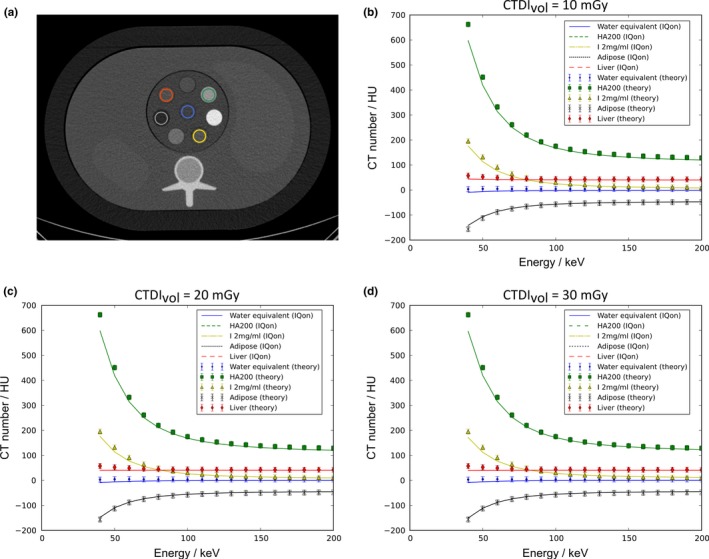
Quantitative measurement of the HU accuracy produced by VMI images of the medium‐sized phantom (350 × 250 mm^2^) at different energy values. (a) shows the phantom with the modular insert together with the selected ROIs for the evaluation. Images were taken for three different dose levels with an approximate CTDI_vol_ of 10 mGy, 20 mGy and 35 mGy as reported by the scanner. For each ROI the spectral attenuation plots produced using the dual‐layer spectral CT viewer are plotted against theoretical values computed for the phantom composition. Overall, good agreement is observed for the CT numbers (b–d) while the largest deviations are obtained at lower energies. The error bars indicate the accuracy by which the theoretical values could be determined regarding density and composition fluctuations of the provided samples.

Besides the improved CNR in VMIs, the quantitative HU values have been determined for various tissue‐equivalent materials at different VMI energy levels. The results of this evaluation is shown graphically in Fig. 6. Overall we obtained a good agreement between measured and theoretical values. The error bars for the theoretical values in Figs. 6(b)–6(d) indicate the accuracy of the calculated reference values. Typical errors of HU values in VMIs are well below 10% for medium and high energies. At energies lower than 60 keV, deviations of measured values from theoretical values can be detected which do not fall into the error intervals. This behavior can partly be attributed to a general problem in a two bin spectral CT, namely the limitation of the decomposition into two basis materials. Especially for elements with higher atomic numbers like calcium or iodine, a two material basis is increasingly inaccurate when considering lower energies.

The determined mean HU are mostly independent on the radiation dose which is again underlining the good quantitative performance of the spectral reconstruction algorithm.

### 3.C Quantitative assessment of iodine concentrations in spectral images:

**Figure 7:**
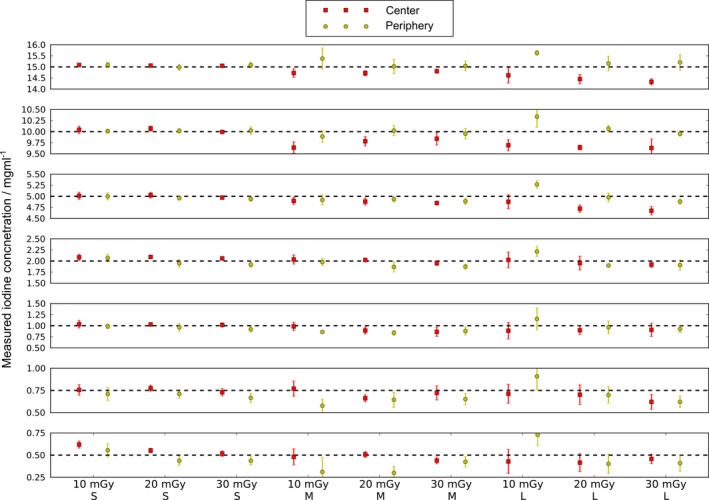
Overview of the quantitative values measured for different iodine concentrations in the range of 0.5 mg/ml–15 mg/ml. The measurements include three different phantom sizes as well as three exposure levels. All concentrations were measured in the center as well as in the periphery of the field‐of‐view. Each individual scan was repeated five times and the error bars indicate associated standard deviations. The mean RMS error of the determined iodine concentrations is in the order of 0.1 mg/ml – 0.3 mg/ml independent on the nominal concentrations.

The quantitative measurement of iodine concentrations is summarized in Fig. 7. The points show the mean and standard deviation as error bars over five CT scans for each investigated exposure level, phantom size, and measurement location within the FOV. The relative bias of these measurements is defined as the absolute difference of the measured mean value $\overset{¯}{c}$ in each ROI from the nominal value $c_{\mathrm{nom}}$, normalized to the nominal value:

$$\mathrm{Rel}.\mathrm{bias}=\frac{\left|\overset{¯}{c}-c_{\mathrm{nom}}\right|}{c_{\mathrm{nom}}}.$$

**Figure 8:**
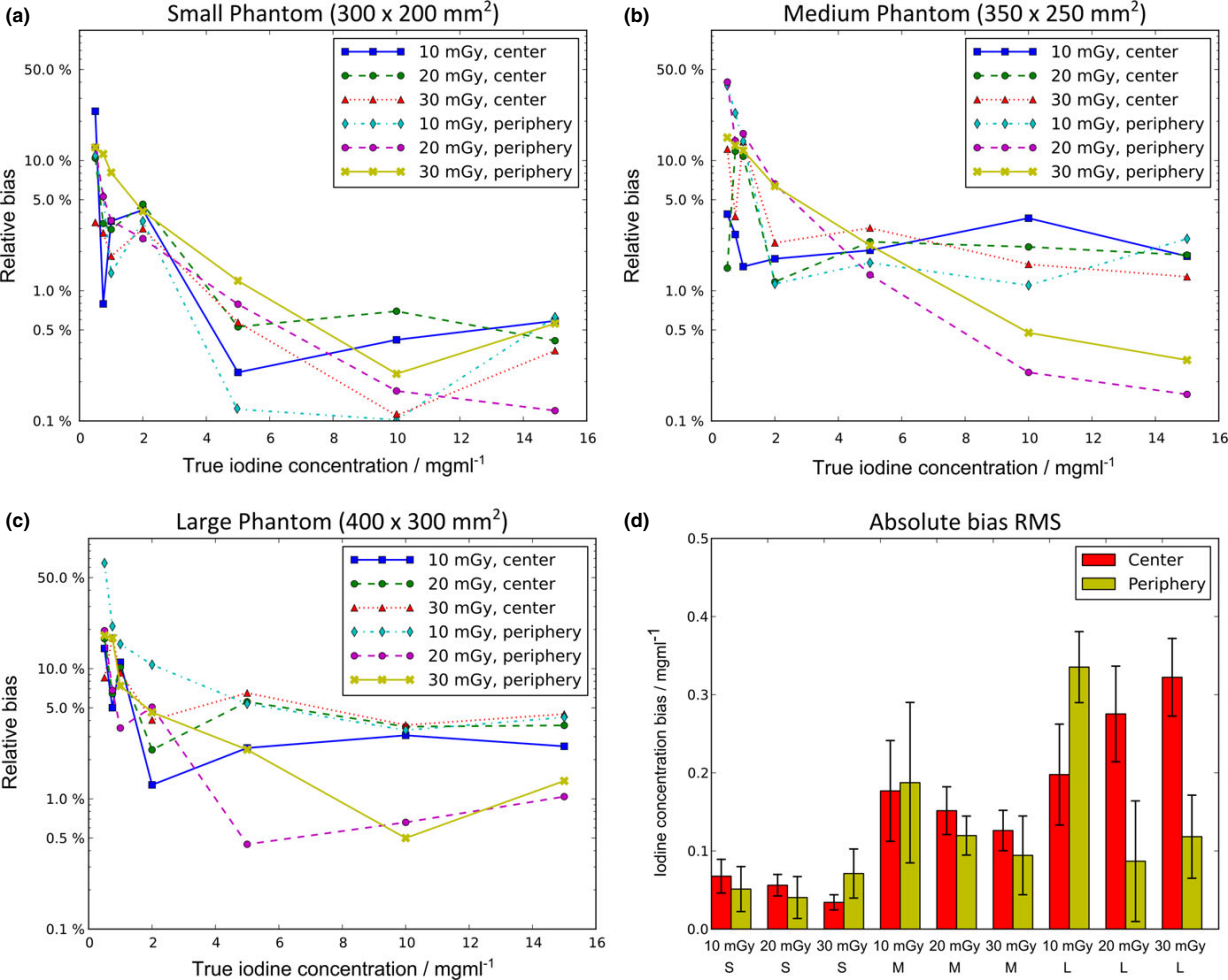
The relative bias of the measured iodine concentrations using the three available phantom sizes in shown in (a–c). For concentrations ≥ 1 mg/ml, the relative bias less than a few percent. For concentrations below 1 mg/ml, the values rise quickly due to the RMS errors in the range of 0.1 mg/ml – 0.3 mg/ml and decreasing nominal values. The distribution of the RMS concentration error across the phantom configurations and exposure levels is shown in (d).

The line‐plots in Figs. 8(a)–8(c) display the relative bias obtained at all three investigated phantom sizes. In all cases the maximum error of the iodine concentration was less than 7% of the nominal values for concentrations larger than 1 mg/ml. Considering iodine concentrations less than 1 mg/ml, the relative bias often extends to values larger than 20%. This behavior can be understood by looking at the results shown in Fig. 8(d) where the RMS of the absolute deviations of the measured concentrations from the nominal values is shown. The average RMS deviation is typically small with values of approximately 0.2 mg/ml. This tendency can also be appreciated in Fig. 7. When normalized to small nominal values below 1 mg/ml, a large relative bias is obtained even if the absolute deviations remain at a low level. Additionally, no false‐positive detection of iodine could be observed in the background. Therefore, the overall presence of iodine is reliably detected even for very low concentrations of 0.5 mg/ml.

To compare the different scenarios investigated, we calculated the average of the relative bias values in each individual phantom configuration, exposure level and measurement position. Due to the limitations discussed above, only concentrations ≥ 1 mg/ml where taken into account. Notably, the mean bias averaged over all presented cases was 3.4%.

For the measurements with the smallest phantom configuration of 300 × 200 mm^2^, the relative bias averaged over all concentrations ≥ 1 mg/ml and all dose levels was 1.7%. The medium‐sized phantom (350 × 250 mm^2^) yielded a slightly larger mean bias of 3.9% and the corresponding value determined in the large configuration covering 400 × 300 mm^2^ was 4.7%. Therefore, a slightly decreased accuracy of the measured iodine concentration is to be expected in larger patients. Between locations in the center of the FOV and the periphery no significant difference in overall performance was observed (3.3% vs. 3.5% mean bias).

Regarding radiation dose, only minor differences in the mean bias values were obtained. Averaged over all scans taken at 10 mGy the bias amounted to 3.5% while the corresponding values for the 20 mGy exposure level was 3.2%. At 30 mGy the mean bias yielded 3.6%.

### 3.D Quantitative assessment of the effective‐Z accuracy:

The spectral accuracy of measured effective‐Z numbers was determined for the tissue‐equivalent materials. The theoretical reference values have been supplied by the phantom manufacturer. For the tissue materials, the values yielded by the CT scans are very close to the theoretical values (Table 3). The mean relative bias calculated over all measured values was found to be 1.9%. The maximum detected bias was 8.2% which was found in the adipose tissue surrogate. Being significantly higher than the other effective Z readings, this is a remarkable result as there is no clear physical explanation for a systematic error. The results for the adipose‐like material in the section investigating the accuracy of energy‐dependent CT numbers indicate a very good agreement between the values obtained with dual‐layer CT and the theoretical values. This observation is also relevant for the calculation of effective Z values as this process can be described be described by a linear combination of spectral basis images for which VMIs are a special case.^40^ Therefore we suspect that the nominal effective atomic number for the adipose surrogate is inaccurately reported by the phantom manufacturer. This assumption can also be backed by the fact that calculating the effective Z number directly using (eq. (5)) and the elemental composition yields 6.27 in case of the adipose surrogate material, which improves the average relative error for this insert to 2.7%.

**Table 3:** Quantitative evaluation of the measured effective‐Z values. The nominal values for reference were supplied by the phantom manufacturer. For all tissue‐equivalent materials with the exception of adipose‐like insert the match between measured and nominal values is typically within 1%. A Bias of up to 8% is exclusively seen in the adipose‐like material, indicating a potentially incorrect reference for this sample

Insert	Nominal eff. Z	CTDI_vol_ = 10 mGy		CTDI_vol_ = 20 mGy		CTDI_vol_ = 30 mGy	
		Measured eff. Z	Relative bias (%)	Measured eff. Z	Relative bias (%)	Measured eff. Z	Relative bias (%)
Water	7.21	7.20	0.1	7.18	0.4	7.18	0.4
Liver	7.32	7.31	0.1	7.25	0.9	7.24	1.1
Muscle	7.31	7.34	0.4	7.27	0.6	7.27	0.6
Adipose	5.65	6.11	8.2	6.04	6.8	6.04	6.8
HA 200	9.76	9.72	0.4	9.70	0.6	9.68	0.8

For most applications, we found quantitative accuracy within 1% of the nominal values when determining effective atomic numbers using the dual‐layer scanner.

## 4 DISCUSSION

In this work, a newly introduced dual‐layer spectral CT scanner has been characterized in terms of its spectral imaging performance. At the investigated iterative processing levels, significantly increased CNR values have been obtained using virtual mono‐energetic images. The largest factor contributing to this result is the reduced noise level in VMIs compared to conventional images which was shown to be largely independent of the energy. This result is especially worth mentioning since noise in VMIs usually has a distinct energy dependency and eventually even exceeds the noise in the conventional images. Therefore, a tradeoff between increased contrast and increased noise toward lower energies must be made in other systems. This generally leads to an optimum energy level for viewing of VMIs.^41^, ^42^ Such limitations can be overcome by the investigated dual‐layer spectral CT scanner due to its proprietary spectral reconstruction algorithm which exploits basis material image noise characteristics. The maximum obtained increase in CNR resulted in a factor of 3.3 at iDose/spectral reconstruction level 0. Together with the observation that the CNR for low contrast tissues and low radiation exposure in VMIs exceeds conventional images, the presented curves encourage an improved detectability of low contrast features in VMIs. This will be of particular importance in low‐dose acquisitions. The curves for high contrast features containing calcium or iodine show significantly different behavior than curves for liver or muscle‐equivalent inserts. This can be explained by the fact that the liver and muscle inserts effectively exhibit a similar spectral behavior of the mass attenuation coefficient compared to the water‐equivalent background. Therefore, only minor energy dependency of contrast is to be expected for these inserts and the CNR curves remain mostly parallel. Increased CNR over the complete energy range can in this case be attributed exclusively to lower noise values in the VMIs. In clinical routine higher levels of iterative processing will also be of interest to further reduce radiation exposure and improve image quality. The choice of the optimal reconstruction parameters is typically highly task‐dependent as features of very different contrast and size are investigated in various diseases. Therefore, subsequent clinical studies will be needed to further evaluate the benefits of VMIs in the diagnosis of various diseases.

The quantification of iodine concentrations generally showed good agreement between nominal and measured values. Until now, the accuracy of iodine concentration measurements using dual‐energy CT has been reported to decrease with larger patient sizes.^43^, ^44^ However, the size‐dependent increase in iodine concentration bias [c.f. Fig. 8(d)] observed with dual‐layer CT is considerably smaller than the values reported so far in work related to other dual‐energy techniques. The iodine quantification results presented in this study generally seem slightly more accurate than previously reported in literature on dual‐layer CT.^45^, ^46^ However, iodine quantification in the dual‐layer scanner is optimized for clinical applications with mixtures of iodine with blood or tissue.^45^ Therefore, such results are difficult to compare directly since the values displayed in iodine concentration maps depend on the background material in which the iodine is embedded. To the best of our knowledge, the present studies on dual‐layer‐based iodine quantification all use different solvents to embed the iodine and therefore might yield a slightly different bias. The combination of improved CNR and reliable iodine concentration measurements in a broad range of patient sizes allows for possibly reduced amounts of contrast media in the clinical routine. This is an important improvement for the clinical routine when considering that a majority of all CT studies involve contrast agents and that iodine, even nonionic iodine, poses risks to patients.^34^, ^47^, ^48^ With respect to side effect for example it is well known that the use of nonionic iodine as a contrast medium can result in acute allergic‐like reactions, ranging from mild symptoms (such as urticaria and itching) to more severe reactions such as cardiopulmonary arrest.^47^ Moreover, we have an aging population, and older patients are more sensitive to contrast load.^48^ Additionally, a significant reduction in cost for the healthcare system can be achieved when reducing the amount of iodine. To determine the ideal iodine concentration and thereby the potential amount contrast medium dose reduction a clinical evaluation need to be performed in the near future. Finally, it should be considered that the manufacturing tolerance of the phantoms itself leads to uncertainties in the nominal iodine concentration values that might also be in order of a few percent. Fortunately though, errors in the range of ± 0.25 mg/ml iodine as presented in Fig. 8(d) will most likely not affect differential diagnosis in clinical context. For example, median iodine concentrations of 2.56 mg/ml vs. 1.35 mg/ml have recently been reported in healthy vs. infarcted myocardial tissue^49^ and similarly, 15.3 mg/ml vs. 19.2 mg/ml for benign vs. malignant pulmonary lesions in the venous phase^50^ where the differences in iodine uptake clearly exceed the quantification bias. Especially the latter case in Ref. 50 demonstrates the potential for contrast medium dose reduction as the studies were performed with administration of 1.5 ml contrast agent per kg body weight. Reducing this amount to a third would in theory still result in a difference of 1.3 mg/ml iodine and would therefore be safe to diagnose.

The translation away from dual‐energy concepts toward spectral detector‐based solutions results in a paradigm shift in the clinical routine, because dual‐layer spectral CT now allows to always acquire spectral information over the full field‐of‐view. This will allow to define additional diagnostic application for spectral imaging. Furthermore, this is a first step toward detector‐based spectral imaging, which will be followed by spectral photon‐counting CT (SPCCT) in the future.^30^, ^51^, ^52^ This future clinical technology promises to overcome major drawback of current scanners with quantitative imaging, material specific (k‐edge) imaging, and a new level of diagnostic image quality in combination with significant reduction in radiation exposure. With respect to this development, dual‐layer spectral CT allows now to start working on the additional diagnostic benefit for current and future systems.

In conclusion, we report on experimental evaluation of a full‐body dual‐layer spectral CT. Our results demonstrate the clinical possibilities when fully utilizing the potentials of spectral imaging. High quantitative accuracy of the various spectral results could be demonstrated in this study. In the near future, dual‐layer spectral CT will enhance the capabilities of CT diagnostics in a wide range of patient groups and diseases.

## CONFLICT OF INTEREST

The authors declare no conflicts of interest.
